# Supplementary material for: Co-Doped CeO2/Activated C Nanocomposite Functionalized with Ionic Liquid for Colorimetric Biosensing of H2O2 via Peroxidase Mimicking
Source: Molecules. 2023 Apr 9;28(8):3325. doi: 10.3390/molecules28083325 (PMC10145388; doi:10.3390/molecules28083325)
Supplement: Supplementary file 1 [file molecules-28-03325-s001.zip › molecules-2295938-supplementary.pdf]

Supplementary Materials

Co Doped CeO_2/Activated C Nanocomposite Functionalized with Ionic Liquid for Colorimetric Biosensing of H_2O_2 via Peroxidase Mimicking

Abdul Khaliq [1], Ruqia Nazir [1], Muslim Khan [1,*], Abdur Rahim [2], Muhammad Asad [1], Mohibullah Shah [3], Mansoor Khan [1], Riaz Ullah [4], Essam A. Ali [5], Ajmir Khan [6] and Umar Nishan [1,*]

1 Department of Chemistry, Kohat University of Science and Technology, Kohat KP 26000, Pakistan
2 Department of Chemistry, COMSATS University Islamabad, Park Road, Islamabad 45550, Pakistan
3 Department of Biochemistry, Bahauddin Zakariya University, Multan 66000, Pakistan; mohib@bzu.edu.pk
4 Department of Pharmacognosy, College of Pharmacy; King Saud University Riyadh Saudi Arabia
5 Department of Pharmaceutical Chemistry; College of Pharmacy; King Saud University Riyadh Saudi Arabia
6 School of Packaging, 448 Wilson Rd, East Lansing, 48824 Michigan State University, MI, USA
* Correspondence: unigraz2012@yahoo.com (M.K.); umarnishan85@gmail.com (U.N.)

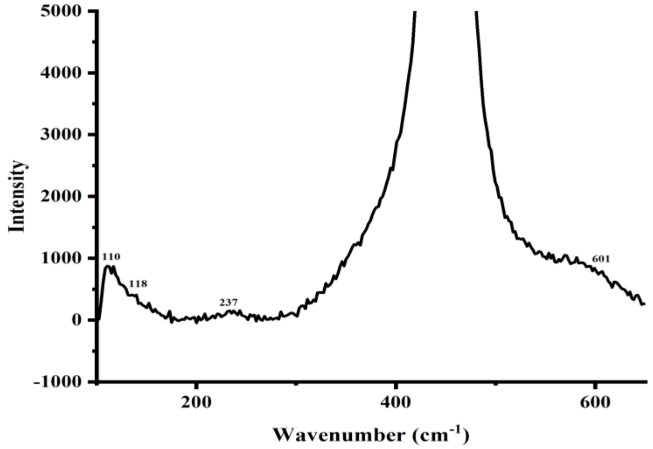

Figure S1. showing the peaks at 110, 118, 237 and 601 cm^{-1}.
